# Walking Like Dinosaurs: Chickens with Artificial Tails Provide Clues about Non-Avian Theropod Locomotion

**DOI:** 10.1371/journal.pone.0088458

**Published:** 2014-02-05

**Authors:** Bruno Grossi, José Iriarte-Díaz, Omar Larach, Mauricio Canals, Rodrigo A. Vásquez

**Affiliations:** 1 Institute of Ecology and Biodiversity, Facultad de Ciencias, Universidad de Chile, Santiago, Chile; 2 Departamento de Ciencias Ecológicas, Facultad de Ciencias, Universidad de Chile, Santiago, Chile; 3 Department of Oral Biology, University of Illinois at Chicago, Chicago, Illinois, United States of America; 4 Department of Organismal Biology and Anatomy, University of Chicago, Chicago, Illinois, United States of America; Raymond M. Alf Museum of Paleontology, United States of America

## Abstract

Birds still share many traits with their dinosaur ancestors, making them the best living group to reconstruct certain aspects of non-avian theropod biology. Bipedal, digitigrade locomotion and parasagittal hindlimb movement are some of those inherited traits. Living birds, however, maintain an unusually crouched hindlimb posture and locomotion powered by knee flexion, in contrast to the inferred primitive condition of non-avian theropods: more upright posture and limb movement powered by femur retraction. Such functional differences, which are associated with a gradual, anterior shift of the centre of mass in theropods along the bird line, make the use of extant birds to study non-avian theropod locomotion problematic. Here we show that, by experimentally manipulating the location of the centre of mass in living birds, it is possible to recreate limb posture and kinematics inferred for extinct bipedal dinosaurs. Chickens raised wearing artificial tails, and consequently with more posteriorly located centre of mass, showed a more vertical orientation of the femur during standing and increased femoral displacement during locomotion. Our results support the hypothesis that gradual changes in the location of the centre of mass resulted in more crouched hindlimb postures and a shift from hip-driven to knee-driven limb movements through theropod evolution. This study suggests that, through careful experimental manipulations during the growth phase of ontogeny, extant birds can potentially be used to gain important insights into previously unexplored aspects of bipedal non-avian theropod locomotion.

## Introduction

Based on multiple lines of evidence, it is now widely accepted that birds evolved from bipedal theropod dinosaurs [1], [2], [3], [4]. Birds have inherited numerous locomotory traits from their dinosaur ancestors, including bipedalism, fully erect posture, and parasagittal hindlimb movement, which are not shared with the other extant group of archosaurs, the crocodilians. Therefore, it is appealing to think of birds as a model system to gain insights into aspects of non-avian dinosaur biology that are hard to study directly from fossil material, such as the relationship between limb morphology, posture, and locomotion [5], [6], [7]. However, non-avian theropods differ from birds in other traits, cautioning the direct use of extant birds to study non-avian theropod locomotion. Some of these differences are related to the evolutionary shift in the location of the centre of mass (CoM) through theropod evolution, from a posteriorly located CoM in non-avian theropods to a more anterior CoM in birds, due to the progressive enlargement of the pectoral limb [8]. For a biped to balance at mid-stance, the feet must be placed directly underneath the CoM, so the location of the CoM is a major factor influencing limb orientation at mid-stance [9]. Consequently, birds have unusually flexed postures at mid-stance, with a highly flexed hip and horizontal femur, and feet placed cranial to the hip. In addition, bird bipedalism is often characterized as ‘knee driven’, where most of the hindlimb movement is achieved by knee flexion powered by strong ‘hamstring’ muscles [5], [10]. In contrast, it has been hypothesized that non-avian bipedal dinosaurs had more vertical femora due to the more posteriorly located CoM, and that their hindlimb movement was ‘hip-driven’, powered mainly by the caudofemoralis longus muscle (CFL). The CFL is a large muscle that extends from the tail to the proximal femur and knee, powerfully retracting the femur, and it is expected to have produced larger femoral range of motion in extinct dinosaurs than in birds [5], [11], [12]. However, despite studies suggesting a strong correlation between changes in morphology and postural and locomotor traits in birds [5], [6], [8], [11], [12], [13], [14], no direct, experimental evidence has yet been found. In fact, only one experimental study, to our knowledge, has attempted to test the relationship between CoM and postural and kinematic changes in birds. In an integrative analysis of posture, limb kinematics and bone loading patterns, Carrano and Biewener [7] attached artificial tails to chickens, thus moving the CoM caudally, hoping to recreate theropod-like limb posture and locomotion. However, their study produced unexpected results: birds with attached tails showed even more horizontally oriented femora, while no qualitative changes in kinematics were observed during locomotion compared to non-manipulated chickens. Here we present a modified study, based on Carrano and Biewener's experiments, in which we attached more realistic artificial tail to chickens shortly after hatching, and allowed proper exercise during ontogeny. We expected adult chickens with added tails to show a more vertical femur in standing position and increased femoral excursion during locomotion as postulated for non-avian theropod dinosaurs.

## Materials and Methods

**Figure 1:**
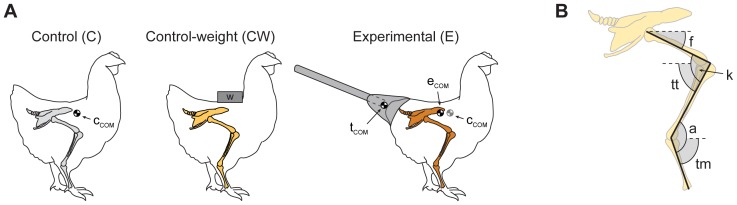
Experimental conditions and kinematic parameters measured. (**A**) Scheme of the control (C, grey hindlimbs), control-weight (CW, yellow hindlimbs), and experimental (E, orange hindlimbs) subjects. Control-weight subjects were raised with extra weight located over the pelvis. Experimental animals were raised carrying a wooden stick inserted in modeling clay and attached to the pelvic girdle. Estimations of the center of mass of the tail rig (t_COM_), as well as of a control (c_COM_) and of an experimental individual (e_COM_), are shown. (**B**) Diagram of the segmental angles (f, femur; tt, tibio-tarsus; tm, tarso-metatarsus) and joint angles (k, knee; a, ankle) used in this study.

Twelve domestic chickens (*Gallus gallus*) were reared from two days after hatching (body mass of 43.0±2.8 g) until they reached sexual maturity (ca. 12 weeks; body mass of 725±51 g), and maintained with food and water *ad libitum*. Subjects were divided into three groups of four subjects each: a control (C), a control-weight (CW) and an experimental (E) group. All birds were of the same age, and there were no differences in final weight between groups. Throughout the experiment, all subjects were housed in a circular enclosure of 1.8 m in diameter and 1.0 m high. To recreate a non-avian theropod configuration, an artificial tail was attached to the rear area of experimental subjects (Fig. 1A and Video S1). The tail was made of a wooden stick (7 mm diameter) inserted in a solid modelling clay base (Fimo clay, Eberhard Faber, Germany), which was adjusted to the shape of each chicken's pelvic girdle, making the stick continuous with the projection of the caudal vertebrae and pygostyle. The clay base was modelled with a conical shape to reproduce the distribution of mass in non-avian theropod tails more realistically, i.e., with most of the tail mass distributed proximally and decreasing to the distal end. The tail was kept in place by attaching the clay base to the body with an elastic fabric coat with Velcro fasteners. The coat and tail were replaced every five days as the chicken grew. The length of the tail was kept closely similar to the length of the chicken's body and ranged from 9 to 28 cm throughout the growth period. Based on previous literature and examination of published reconstructions [5], [15], [16], [17] we used a tail mass weighing 15% of the chicken mass, which was probably close to the tail/body mass proportion for smaller theropod dinosaurs [5], [16]. The location of the CoM of the experimental tail was calculated by taking pictures of the tail rig hanging from a wire in different positions. The change in the location of the animal's CoM was expected to move posteriorly 15% of the distance of the tail's CoM to the expected location of the CoM in a normal, control subject. The location of the CoM in a control subject was assumed to be vertically aligned with the middle of the subject's foot during standing (Fig. 1A). In an average adult chicken, the experimental tails was expected to displace the animal's CoM posteriorly around 2 cm. To control for postural and kinematics changes produced purely by an increase in supported weight, a control-weight group was defined in which body mass was increased by the same amount as in the experimental group, but instead of adding an experimental tail, a lead mass was attached to the coat above the pelvic girdle, as close as the CoM as possible. We paid special attention to allow experimental subjects to exercise continuously. Therefore, control-weight subjects wore the coat with the lead mass, and experimental subjects wore the coat and the tail all the time, and the social rearing conditions allowed continuous activity (i.e., locomotor exercise and social interactions) of all the subjects. No ill effects or distress were observed in neither experimental nor control-weight subjects. Indeed, subjects got used to the coats and artificial tails and behaved as normal. All procedures followed the norms of and were approved by the Bioethics and Animal Use Committee at the University of Chile.

At 12 weeks of age, all subjects were videotaped laterally with a digital video camera (SONY digital 8 DCR-TRD 330) at 30 frames per second, in two conditions: while standing quietly (chickens standing without moving for at least 10 s) and while walking spontaneously along the length a 3 m track motivated by food on one side of the track. In both conditions, each subject was recorded four times. The mean of each subject was used for statistical analysis. In the locomotion trials, speeds ranged between 0.4 and 0.6 m/s for both groups. Since we were interested in assessing the influence of artificial tails on femoral movement during locomotion, the use of a narrow range of speeds was important because it is known that limb movement can change with speed in birds [18]. In each subject, plucked areas of the right side of the hip, knee, ankle, and metatarsal-phalangeal joints were marked with coloured adhesive tape or black ink for analysis. Videotaped frames were digitized using SigmaScan Pro 5 (SPSS Inc.), and segmental and joint angles were measured (Fig. 1B). Segmental angles were defined as the angle between a limb segment (i.e., femur, tibiotarsus and tarsometatarsus) and the horizontal and joint angles were defined as the angle between two limb segments (Fig. 1B). The stance phase was defined as the period of time in which the foot is contact with the ground, from foot-down (i.e., the first frame in which the foot touches the ground) to toe-off (i.e., the first frame in which the foot was off the ground), identified from the videos.

To estimate the cross-sectional characteristics of the femur, frontal and lateral projections x-rays images were taken using 50 kV and 300 mA for 0.02s. A focus-film distance of 1 m was used to avoid image and size distortion. Medio-lateral (ML) and antero-posterior (AP) second moment of area of the femur at midshaft were estimated as *I*_ML_ = π(*D* ^3^_AP_ *D*_ML_−*d* ^3^_AP_ *d*_ML_)/64 and *I*_AP_ = π(*D*_AP_ *D* ^3^_ML_−*d*_AP_ *d* ^3^_ML_)/64, respectively, where *D* corresponds to the external cortical diameter of the femur and *d* corresponds to the internal cortical diameter of the femur. The polar moment of area (*J*) was calculated as *J* = *I*_ML_+*I*_AP_.

Differences among groups were tested with one-way ANOVAs and post-hoc comparisons were performed using Tukey's *t*-tests. Significance was assessed with α = 0.05.

## Results

**Figure 2:**
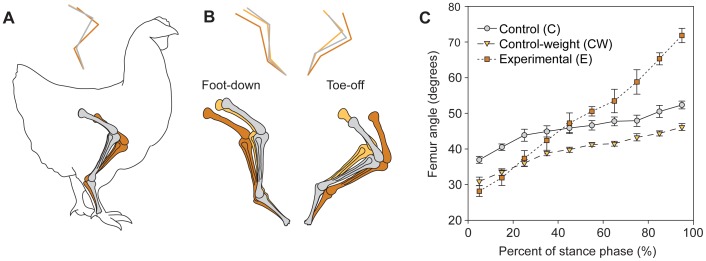
Effect of added mass and experimental tail on limb posture and kinematics. (**A**) Diagram showing the average limb posture during standing position of control (C), control-weight (CW), and experimental subjects (E). The stick figure above indicates the limb segment orientation among groups to visualize postural differences among treatments. Hindlimb bones and segment orientation are color-coded as in Fig. 1. (**B**) Diagram of the average limb posture during touch down (beginning of support phase) and during lift-off (end of support phase) of control, control-weight, and experimental animals. (**C**) Femur angle through the support phase for control, control-weight, and experimental subjects. Data are presented as mean ± s.e.m.

In standing position, experimental subjects showed a limb posture with a more vertically oriented femur and a more horizontally oriented tibiotarsus, due to a more flexed ankle joint (Table 1 and Fig. 2A). During slow walking, significant differences in kinematics were observed among treatments (Table 2, Fig. 2B, and Video S1). At the end of the stance phase, the knee joint was more extended in the experimental group (102.0±2.1 deg) than in the control group (83.3±6.0 deg). This resulted in reduced range of knee flexion during the stance phase in the experimental subjects compared to the control group (E: 30.1±3.4 deg; C: 41.3±3.1 deg). The ankle joint of experimental subjects was also more extended than that of the control group at both the onset (E: 138.7±2.1 deg; C: 128.8±2.6 deg) and offset of the stance phase (E: 152.4±1.9 deg; C: 136.0±4.9 deg). Limb segmental angles also showed differences among treatments. Of all the limb segments, the femur showed the largest difference between control and experimental conditions (Table 2). In experimental subjects, the femur was more protracted at the beginning of the stance phase and more retracted at the end of the stance phase than subjects in the control group (Fig. 2B,C). As a consequence, the femoral range of motion of experimental subjects during the stance phase was almost three times larger than that of control subjects (E: 43.7±0.8 deg; C: 15.4±0.5 deg).

**Table 1:** Joint and segmental angles (mean±s.e.m.) during standing position for control, control-weight and experimental birds.

		Group			ANOVA	
		Control	Weight-control	Experimental	*F*_2,10_	*P*
**Joint angles**	**Knee**	88.5±2.0	89.5±2.7	83.1±4.7	1.12	0.36
	**Ankle**	133.4±1.8^a^	131.5±1.9^a^	110.4±6.4^b^	11.4	0.003
**Segmental angles**	**Femur**	27.1±1.0^a^	28.0±1.2^a^	38.3±0.5^b^	38.9	<0.0001
	**Tibiotarsus**	61.2±1.7^a^	61.2±2.6^a^	44.6±5.0^b^	8.59	0.007
	**Tarsometatarsus**	71.6±1.4	70.8±3.0	65.7±1.4	2.5	0.13

Different letters represent significant differences among groups based on Tukey post-hoc comparisons (α = 0.05).

**Table 2:** Joint and segmental angles (mean ± s.e.m.) during slow walking for control, control-weight, and experimental birds.

			Group			ANOVA	
			Control	Weight-control	Experimental	*F*_2,9_	*P*
**Joint Angles**	**Knee**	**Start**	124.9±2.9	128.2±1.6	132.1±1.2	1.95	0.2
		**End**	83.3±6.0^a^	82.5±1.8^a^	102.0±2.7^b^	6.69	0.017
		**Range**	41.3±3.1^a^	45.8±2.1^a^	30.1±3.4^b^	7.79	0.011
	**Ankle**	**Start**	128.8±2.6^a^	138.0±2.1^b^	138.7±2.1^b^	5.73	0.025
		**End**	136.0±4.9^a^	146.8±0.8^a,b^	152.4±1.9^b^	7.32	0.013
		**Range**	7.2±2.5	8.8±2.0	13.8±1.5	2.83	0.11
**Segmental Angles**	**Femur**	**Start**	36.9±1.0^a^	30.9±1.2^b^	28.6±0.7^b^	21	<0.0001
		**End**	52.3±1.1^a^	46.1±1.0^b^	71.8±1.0^c^	172.9	<0.0001
		**Range**	15.4±0.5^a^	15.2±1.5^a^	43.7±0.8^b^	258.7	<0.0001
	**Tibiotarsus**	**Start**	88.7±1.4^a^	94.7±1.8^a^	102.4±1.8^b^	16.2	0.001
		**End**	26.6±3.2^a^	40.2±2.3^b^	28.9±2.2^a^	7.93	0.01
		**Range**	62.1±1.9^a,b^	54.5±3.3^a^	73.5±2.9^b^	10.4	0.005
	**Tarsometatarsus**	**Start**	42.1±1.3^a,b^	44.8±1.7^a^	36.3±2.6^b^	4.96	0.035
		**End**	108.2±2.6^a^	108.8±3.1^a^	123.6±0.6^b^	13.6	0.002
		**Range**	66.2±2.8^a^	64.0±4.5^a^	87.3±3.2^b^	12.8	0.002

Different letters represent significant differences among groups based on Tukey post-hoc comparisons (α = 0.05).

It is possible that postural and kinematic changes observed in experimental subjects were the result of increased weight and not change in CoM location. However, no postural changes were observed between the control-weight group and the control group during standing (Fig 2A and Table 1). During slow walking, the results are a bit more complex. At the beginning of stance phase, knee angle and femur orientation were significantly different in both the control-weight and experimental groups with respect to the control group, suggesting that the added mass of tail was responsible for the kinematic changes. For all other joint and segmental angles, the control-weight group showed either no changes with respect to the control group (e.g., knee angle) or the changes were opposite to the changes observed in the experimental group (Table 2 and Fig. 2B). For example, femur orientation in the control-weight group was consistently more horizontal than in the control group through the stance phase (Fig. 2C), but with similar amount of range of motion (C: 15.4±0.5 deg; CW: 15.2±1.5 deg).

No differences among groups were found in neither antero-posterior (AP) nor medio-lateral (ML) femoral cross-sectional geometry (Table 3). Femoral length, however, tended to be larger in the experimental group than in the control groups, but this difference was only marginally significant (*p* = 0.057; Table 3).

**Table 3:** Morphological parameters of the femur (mean ± s.e.m.) for control, control-weight, and experimental animals.

	Group			ANOVA	
Variable	Control	Control-weight	Experimental	*F*_2,8_	*P*
***L*** ** (mm)**	58.3±1.3	59.8±5.1	62.3±1.5	4.19	0.057
***I*** **_ML_ (mm^4^)**	59.8±17.1	62.2±5.1	82±19.2	2.30	0.16
***I*** **_AP_ (mm^4^)**	58.7±18.7	67.2±18.1	83.4±10.4	1.86	0.21
***J*** ** (mm^4^)**	118.5±35.3	129.4±19.2	165±28.8	2.45	0.15
***I*** **_ML_/** ***L*** **^4^ (×10^−6^)**	5.1±1.0	4.9±0.9	5.4±1.1	0.20	0.82
***I*** **_AP_/** ***L*** **^4^ (×10^−6^)**	5.0±1.2	5.5±2.5	5.6±1.0	0.10	0.90
***J*** **/** ***L*** **^4^ (×10^−6^)**	1.0±2.2	1.0±3.3	1.1±1.8	0.09	0.92

## Discussion

We have shown that the addition of an artificial tail during ontogeny can produce postural and locomotory changes in chickens, consistent with the posture and kinematics inferred for non-avian dinosaurs [5], [6], [11]. The posterior displacement of the CoM produced a more vertically oriented femur during standing (femur in experimental animals was 40% more vertical than control subjects), and increased femoral retraction and decreased knee flexion during walking. These results indicate a shift from the standard bird, knee-driven bipedal locomotion to a more hip-driven locomotion, typical of crocodilians (the only other extant archosaur group), mammals, and hypothetically, bipedal non-avian dinosaurs. These postural and kinematics changes cannot be attributed to an increased weight as subjects of the control-weight group did not show the same changes as the experimental group. In fact, the control-weight subjects showed a more horizontally oriented femur during walking with respect to the control group, similar to that observed in Carrano and Biewener's experimental subjects [7]. Therefore, we conclude that the location of the CoM can be a key factor in defining limb posture and kinematics. It has been proposed that the relative mass of the CFL can be used as a proxy to estimate the relative importance of femoral retraction during locomotion in extinct bipedal dinosaurs [8]. Our data show that for a given CFL mass, femoral retraction can be greatly affected by the location of the CoM and limb postures. Furthermore, limb retraction can be markedly modulated with speed [5], suggesting caution when using simple morphological parameters to estimate functional relationships.

Differences in limb orientation can produce substantial differences in loading regimes on limb bones. The orientation of each limb element to the ground reaction force (GRF) indicates the relative contribution of axial and bending forces to external bone loading: a bone perpendicular to the GRF is expected experience greater bending forces than one parallel to the GRF. Because bone adapts to its loading environment [19], [20], [21], geometric information from limb bones, such as lengths and cross-sectional geometry, are expected to reflect differences in loading regimes and consequently in behavior and locomotor patterns [22], [23]. In this framework, scaling differences in femoral geometry between non-avian theropods and birds have been suggested to be the result of postural differences between these groups [6], [23]. Birds have relatively shorter, stouter femora than non-avian theropods, presumed to be associated with more horizontal orientation. Experimental manipulations of femoral orientation in chickens suggest that torsional loads increase as the femur becomes more horizontal [7] supporting the idea that postural differences could be reflected in differences in limb cross-sectional geometry. To test if the postural differences observed in this study produced changes in limb morphology, we measured the length and mid-shaft cross-sectional properties of the femur in all our individuals. However, we found no differences in cross-sectional femoral geometry among groups. Maybe this is not surprising considering that a recent study analyzing the relationship between posture and femur cross-sectional properties failed to find differences between birds and non-avian theropods [24], suggesting that simple morphological correlates of limb posture should be used with caution. Interestingly, femur length tended to be greater in the experimental group than in both the control-weight and the control group (by 4 and 7%, respectively), although not signifcant. Longer limbs are expected to experience larger bending and torsional moments, so the fact that experimental animals had longer femora suggests that limb verticalization reduces these moments by orienting the bone more parallel to the GRF line of action. If this were the case, it would support the idea that non-avian theropods have relatively thinner femora than extant birds because of postural differences [6].

The present study was inspired by Carrano & Biewener [7] but our results differed markedly from theirs. We suggest that the different outcomes are due to the distinct rearing and exercising conditions used in each study, in addition to the different artificial tails used. First, our experimental subjects lived in a large enclosure under conditions that allowed them to exercise all day long. In Carrano & Biewener's study, experimental chickens were housed individually in smaller cages and were only allowed to exercise 20 minutes per day, 3 days per week, from the 6^th^ to the 12^th^ week. Second, in their study, a lead mass was attached at the distal end of the experimental tail, probably generating excessive displacement of the CoM. During avian evolution, the loss of the CFL and reorganization of the pelvic musculature [5], [13] could have made birds unable to properly carry a postacetabular mass equivalent to that carried by non-avian theropods [5], [6]. In our experimental setup, we attempted to more closely mimic non-avian theropod tail morphology, in which mass is distributed through a distally tapering tail. In addition, we reduced the total tail mass to 15% body mass from the 20% body mass used by Carrano and Biewener. Thus, our study seems to have generated a more gradual and less pronounced change in the moment of inertia produced by the artificial tail, allowing experimental subjects to adjust to the posterior mass by adopting a more vertical position of the femur while standing. Interestingly, the femur kinematics during walking in our control-weight group resembles the results reported in the experimental subjects of Carrano and Biewener. This suggests that their results could be partially explained as a response to the increased loading rather than to the displacement of the CoM.

Due to the phylogenetic relatedness, extant birds have been used to inform functional aspects of non-avian dinosaur locomotion. However, substantial differences in hindlimb morphology between these groups make difficult to assess the validity of inferences obtained from such studies. It has even been proposed that, due to functional convergence, mammals might be a better system to study bipedal dinosaur locomotion [7], [23], but the results reported here show that important aspects of non-avian theropod locomotion can be experimentally recreated in modern birds. One caveat, however, is that our approach uses tail reduction as the mechanism for CoM displacement despite it has been recently shown that the evolutionary change in CoM position was driven instead by forelimb enlargement [8]. Nonetheless, this does not mean that tail reduction had no effect on CoM displacement, but that it was not the most important factor. Ideally we would have increased tail mass and reduced pectoral limb mass but, unfortunately, this is not experimentally feasible. We argue that our experimental approach, although not perfect, was effective in displacing the CoM and recreating locomotor patterns expected in non-avian theropods. Thus, we expect that careful phenotypic manipulation of extant birds can open new avenues of experimental investigation into unexplored facets of dinosaur locomotor mechanics and energetics, providing a more nuanced understanding of the relationship between form and function in dinosaur evolution.

## Supporting Information

Video S1 **Lateral view of a control and an experimental chicken during normal walking.**
(MOV)
